# cJun N-terminal kinase (JNK) phosphorylation of serine 36 is critical for p66Shc activation

**DOI:** 10.1038/srep20930

**Published:** 2016-02-12

**Authors:** Sana Khalid, Astrid Drasche, Marco Thurner, Martin Hermann, Muhammad Imtiaz Ashraf, Friedrich Fresser, Gottfried Baier, Leopold Kremser, Herbert Lindner, Jakob Troppmair

**Affiliations:** 1 Daniel Swarovski Research Laboratory, Department of Visceral, Transplant and Thoracic Surgery, Medical University of Innsbruck, Innsbruck, Austria; 2 Department of Anesthesiology and Critical Care Medicine, Medical University of Innsbruck, Innsbruck, Austria; 3 Department for Pharmacology and Genetics, Division of Translational Cell Genetics, Medical University of Innsbruck, Innsbruck, Austria; 4 Division of Clinical Biochemistry, Protein Micro-Analysis Facility, Biocenter, Medical University of Innsbruck, Innsbruck, Austria

## Abstract

p66Shc-dependent ROS production contributes to many pathologies including ischemia/reperfusion injury (IRI) during solid organ transplantation. Inhibiting p66Shc activation may provide a novel therapeutic approach to prevent damage, which is poorly managed by antioxidants *in vivo*. Previous work suggested that pro-oxidant and a pro-apoptotic function of p66Shc required mitochondrial import, which depended on serine 36 phosphorylation. PKCß has been proposed as S36 kinase but cJun N-terminal kinases (JNKs) may also phosphorylate this residue. To simulate the early stages of ischemia/reperfusion (IR) we either used H_2_O_2_ treatment or hypoxia/reoxygenation (HR). As during reperfusion *in vivo,* we observed increased JNK and p38 activity in mouse embryonic fibroblasts (MEFs) and HL-1 cardiomyocytes along with significantly increased p66ShcS36 phosphorylation, ROS production and cell damage. Application of specific inhibitors caused a pronounced decrease in p66ShcS36 phosphorylation only in the case of JNK1/2. Moreover, S36 phosphorylation of recombinant p66Shc by JNK1 but not PKCß was demonstrated. We further confirmed JNK1/2-dependent regulation of p66ShcS36 phosphorylation, ROS production and cell death using JNK1/2 deficient MEFs. Finally, the low ROS phenotype of JNK1/2 knockout MEFs was reversed by the phosphomimetic p66ShcS36E mutant. Inhibiting JNK1/2-regulated p66Shc activation may thus provide a therapeutic approach for the prevention of oxidative damage.

Physiological levels of reactive oxygen species (ROS) are important for the maintenance of cellular homeostasis while excessive production causes aberrant signaling, inflammasome activation, cell death and ultimately organ damage, which leads to many pathological conditions ranging from diabetes, cancer, atherosclerosis, neurodegenerative diseases, rheumatoid arthritis to ischemia/reperfusion injury (IRI) during solid organ transplantation^1^,^2^,^3^. In transplantation ROS production during early reperfusion is a critical initiating event for the development of IRI, while subsequent inflammation together with altered innate and adaptive immune responses contribute to damage amplification^3^. Several therapeutic approaches are currently being implemented, which mainly target these later events^3^, while efforts to prevent direct detrimental ROS effects through the use of anti-oxidants resulted in no clinical benefit^4^,^5^. Promising novel approaches for limiting or avoiding oxidative damage may come from the suppression of ROS production by targeting the crosstalk between cytoplasmic signaling and mitochondria. Diverse signaling molecules respond to ischemia/reperfusion (IR) including mitogen-activated protein kinases (MAPKs)^6^,^7^,^8^, NF-κB^9^, JAK/STAT^10^,^11^, PI-3 kinase/protein kinase B (PKB/AKT), Pim-1^12^,^13^ or Toll like receptors (TLRs). Evidence for a link between intracellular signaling and the regulation of mitochondrial ROS production has been provided e.g. for p53^14^,^15^,^16^, PKA^17^,^18^, mTOR^19^ or PKCε^20^. Our own work demonstrated prooxidant and pro-apoptotic functions for the MAPK p38 during hypoxia/reoxygenation (HR) and IR^21^,^22^, while signaling through RAF-MEK-ERK protected against mitochondrial accumulation of ROS/Ca^2+^ and cell death^23^,^24^. p66Shc, the longest form of the adaptor proteins of the ShcA family^25^, which normally function in coupling of receptor tyrosine kinase (RTK) stimulation to the recruitment of small G proteins, possesses oxidoreductase activity^26^. p66Shc plays an important role in the generation of mitochondrial ROS^26^ and in the Langendorff-perfused heart p66Shc ablation has been shown to prevent IRI with the same efficiency as antioxidants^27^. Moreover, p66Shc-derived ROS are involved in many pathological conditions and diseases^28^,^29^. ROS p66Shc may be a promising candidate for therapeutic intervention: its activation in the cytosol is controlled by signaling proteins, which respond to cellular stress, p66Shc directly causes mitochondrial ROS production and cell death, and presence of survival signals and normoxic conditions precludes p66Shc activation. Most importantly absence of p66Shc does not affect physiological ROS signaling as evidenced by the normal development and post-natal life of p66Shc-deficient mice^26^.

Although no inhibitors of p66Shc’s oxidoreductase activity are available, understanding the complex mode of p66Shc activation will provide suitable targets for therapeutic interference. PKCß phosphorylation of serine 36 has been implicated in the mitochondrial import of p66Shc, ROS production and cell death induction^30^. Inspection of the amino acid sequence surrounding S36 located in the collagen-homology domain (CH2) domain, which is unique for p66Shc but not present in p52/p46Shc^31^, suggests phosphorylation by MAPKs, e.g. JNK, rather than by PKCs^32^,^33^,^34^,^35^,^36^. Presence of PKC phosphorylation sites is suggested for the phosphotyrosine binding domain of p66Shc^32^. This is also supported by our findings showing that while PKCß inhibition or knockout impaired ROS production it did not affect p66ShcS36 phosphorylation (Haller, Khalid *et al.* manuscript in preparation). Prooxidant function has also been suggested for signaling through JNK1/2^37–41^ and phosphorylation of S36 of p66Shc by JNK has been reported following UV-irradiation^38^ or diallyl trisulfide (DATS) treatment^37^. JNK translocation to the mitochondria was required for ROS generation during anisomycin- or IR-induced stress^40^,^41^. Incubation of human aortic endothelial cells with oxidized low-density lipoprotein (oxLDL) resulted in the phosphorylation of p66Shc on S36 through a pathway involving PKCß upstream of JNK^39^. JNKs are activated during early reperfusion around the time when ROS levels increase^21^,^22^.

In the work presented here we thus systematically addressed a possible role of JNK in controlling the activation of p66Shc, ROS production and cell death in a setting close to ischemia and early reperfusion. Our experiments demonstrate that JNK1/2 regulate p66Shc S36 phosphorylation and mitochondrial ROS production under the conditions studied here and blocking this molecular route may provide a therapeutic mean to prevent cellular damage and death under oxidative stress.

## Results

### Oxidative stress and hypoxia/reoxygenation (HR) lead to activation of JNK/p38 and increased p66ShcS36 phosphorylation

**Figure 1:**
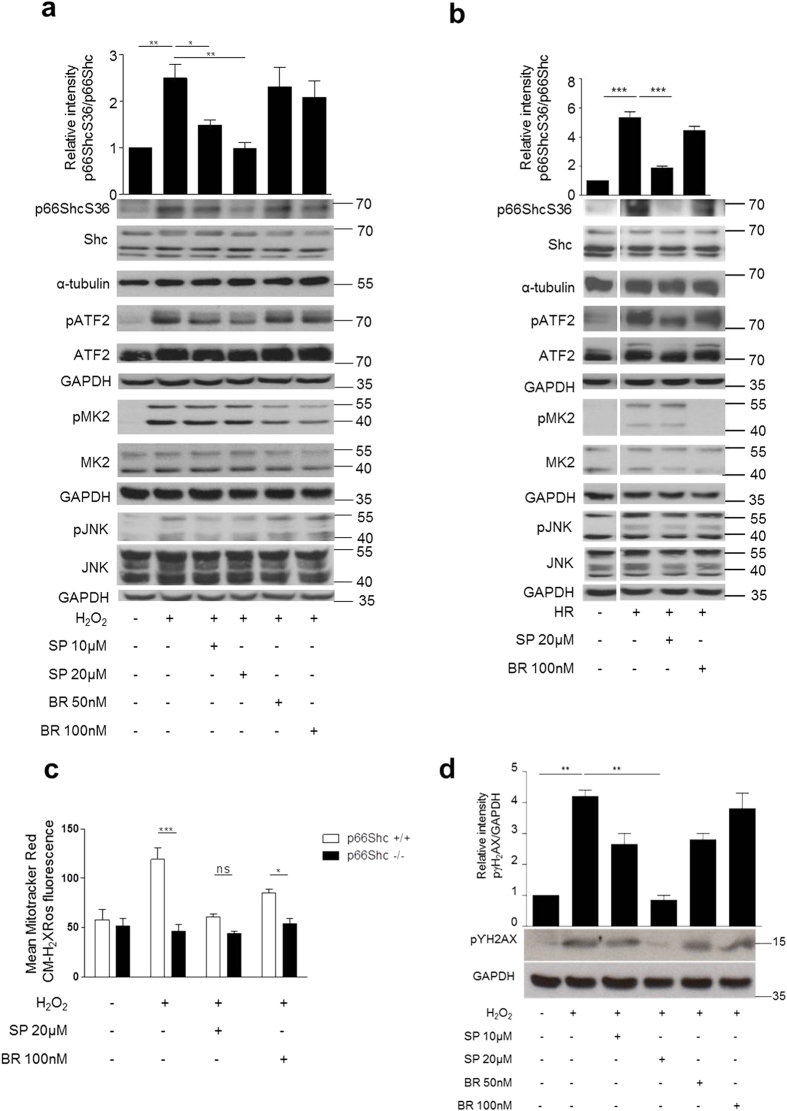
Inhibition of JNK kinases prevents p66ShcS36 phosphorylation under oxidative stress. MEFs were treated with H_2_O_2_ (**a**) (1 mM, 30 min) or exposed to HR (hypoxia 90 min, reoxygenation 15 min) (**b**). Cell lysates were probed with the antibodies indicated to assess p66Shc phosphorylation at S36. Representative Western blots (individual experiment performed under the same experimental conditions and run on the same gel) and summary graphs from at least three independent experiments are shown. Signal intensity of control samples was arbitrarily set at 1. Mitochondrial ROS production was measured in p66Shc^+/+^ and p66Shc^−/−^ MEFs after prooxidant treatment as described in Material and Methods (n ≥ 4) (**c**) and phospho γH2AX was detected by immunoblotting (**d**, lower panel) and summary graph is shown in above panel. SP: SP600125, JNK inhibitor, and BR: BR796, p38 inhibitor were applied 1 h prior to stress application. Original blot shown in (**b**) have been cropped and the full length blots are shown in the Supplementary Figure S1b Statistical analysis was performed using *t*-test or ANOVA (*p < 0.05 **p < 0.01, ***p < 0.001).

Mouse embryonic fibroblasts (MEFs) were exposed to two different treatments commonly used to mimic ischemia/reperfusion (IR), prooxidant treatment with H_2_O_2_ and HR. HR reproduces the early events of ischemia/reperfusion, where mainly the changes in oxygen and nutrient provision are the key triggers for cellular responses. Using phosphorylation-specific and total protein antibodies for p66Shc and JNK1/2 as well as for the p38 substrate MAPKAP kinase 2 (MK2) and the JNK1/2 target ATF2 changes in signaling were analyzed. As shown in Fig. 1a,b and reported previously for similar *in vitro* and *in vivo* settings^21^,^22^ both stimuli resulted in increased activation of JNK and p38, as demonstrated by the phosphorylation of the substrates ATF2 and MK2 (Fig. 1a,b; Supplementary Figure S1a,b), respectively. In addition, we also observed enhanced p66ShcS36 phosphorylation. To identify the kinase phosphorylating p66ShcS36 we employed low molecular weight inhibitors of p38 and JNK, which showed the expected effects on the phosphorylation of their respective targets (MK2 and ATF2 respectively). While pretreatment with the p38-specific inhibitor BIRB796 only marginally inhibited S36 phosphorylation, in the case of JNK1/2 inhibition, p66ShcS36 phosphorylation was almost completely blocked (Fig. 1a,b). We also confirmed JNK1/2-mediated p66ShcS36 phosphorylation regulation upon sIR in cardiomyocyte cell line HL-1 (Supplementary Figure S2a).

### JNK1/2 are required for ROS production

Studies in the Langendorff-perfused heart had demonstrated the requirement for p66Shc in the development of IRI^42^. Blockade of p66Shc phosphorylation by JNK1/2 inhibitor prompted us to investigate whether JNK1/2 may also have a role in inducing prooxidant stress. To this end MEFs were treated with H_2_O_2_ and the effect on mitochondrial ROS production was monitored using MitoTracker Red CM-H2XRos. As shown in Fig. 1c H_2_O_2_ application resulted in increased ROS production, which was suppressed by JNK as well as by the p38 inhibitor. Similarly, HL-1 cells also showed reduced ROS production under sIR in presence of JNK inhibitor (Supplementary Figure S2b).

### JNK1/2 inhibition reduces early DNA damage upon oxidative stress

ROS-induced double strand breaks (DSBs) are characterized by the phosphorylation of the histone γH2AX, which can be monitored by appropriate antibodies^43^. We used this parameter to determine damage upon oxidative stress in MEFs in presence of p38 and JNK inhibitors. As demonstrated in Fig. 1d increased phosphorylation of γH2AX upon H_2_O_2_ treatment was suppressed by the JNK but not by the p38 inhibitor. The same protective effect of JNK inhibition was also observed in HL1 cells upon sIR (Supplementary Figure S2c).

### Prooxidant and HR-induced p66ShcS36 phosphorylation and ROS production are impaired in JNK1/2 knockout cells

**Figure 2:**
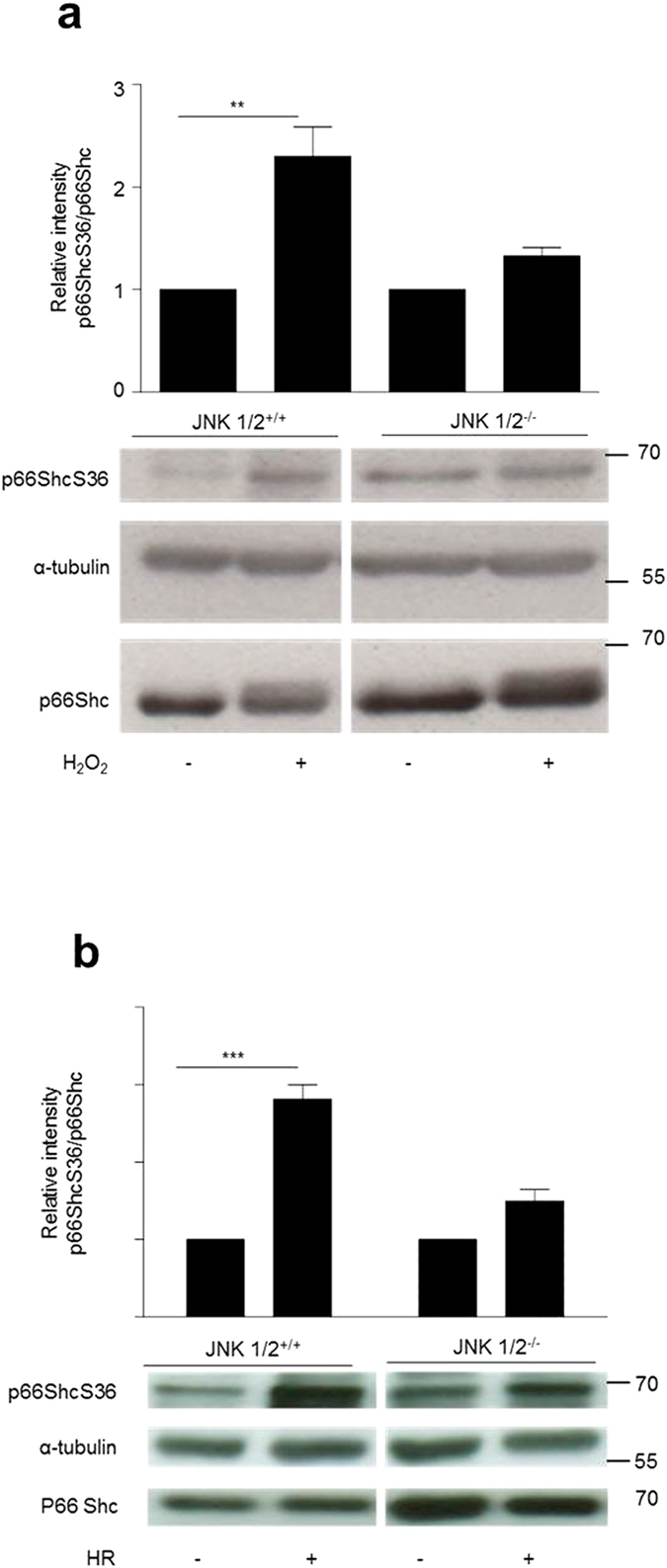
JNK1/2 deficiency results in an impaired p66ShcS36 phosphorylation upon HR and prooxidant treatment. JNK1/2^+/+^ and JNK1/2^−/−^ MEFs were treated with H_2_O_2_ (1 mM, 30 min) (**a**) or HR (hypoxia 90 min, reoxygenation 10 min) (**b**). Cell lysates obtained from the same experiment and run on a single gel were subjected to the analysis for p66Shc S36 phosphorylation by immunoblotting and representative blots are shown. Summary graphs of results obtained in at least three independent experiments are provided. The band intensity observed in control samples was arbitrarily set to 1. Original blots have been cropped and full length blots are shown in the Supplementary Figure S4. Statistical analysis was performed using *t*-test (**p < 0.01, ***p < 0.001).

**Figure 3:**
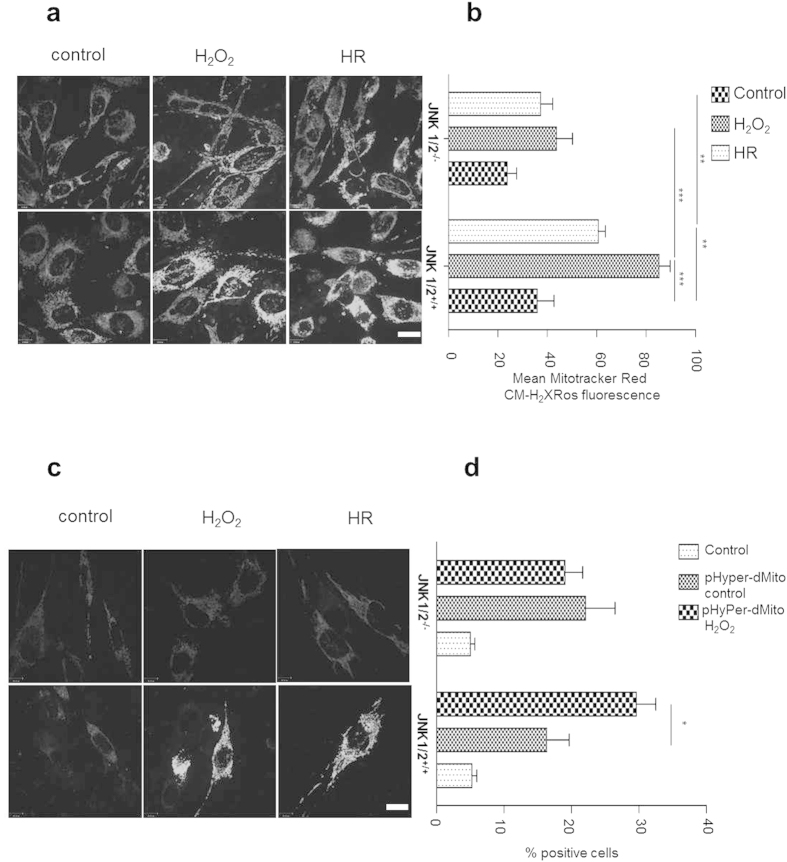
JNK regulates mitochondrial ROS production. JNK1/2^+/+^and JNK1/2^−/−^ MEFs were subjected to H_2_O_2_ (1 mM, 30 min) or exposed to HR (hypoxia 90 min, reoxygenation 10 min). Mitochondrial ROS were either measured after staining the cells with MitoTracker Red CM-H_2_XRos fluorescent dye and analyzed by fluorescence microscopy (panel (**a**) sample Image, panel (**b**) summary graph) or cells indicated were infected with lentiviral particles harboring either pHyPer-Cyto or pHyPer-dMito expression vectors. HyPer fluorescence was analyzed using confocal microscopy (**c**). Quantitative analyses were done by FACS where uninfected cells were used as background control (**d**). Each experiment is repeated at least three times. Size bar = 10 μM. Statistical analysis was performed using *t*-test or ANOVA (*p < 0.05, **p < 0.01, ***p < 0.001).

To corroborate our JNK inhibitor data we employed mouse embryonic fibroblasts deficient in JNK 1, 2. (Supplementary Figure S4a). We observed a higher p66Shc phosphorylation as well as the expression of p66Shc in JNK1/2-deficient MEFs under basal conditions (Fig. 2). But upon treatment with H_2_O_2_ (Fig. 2a) or using HR (Fig. 2b), JNK-deficient MEFs failed to display any noticeable increase in p66ShcS36 phosphorylation, which was pronounced in wild type MEFs. Since p66Shc has been linked to ROS production and serine 36 has been identified as a key residue for this process we also tested the effect of JNK ablation on ROS production under these two conditions. As shown in Fig. 3a,b deletion of JNK1/2 dampened ROS production by these cells. Decrease in the ROS production upon JNK ablation was further validated using mitochondria-targeted protein-based ROS sensor thus confirming a link between JNK1/2 and mitochondrial ROS production (Fig. 3c,d).

### Low mitochondrial ROS phenotype of JNK^−/−^ MEFs is reversed by overexpression of p66ShcS36E mutant protein

**Figure 4:**
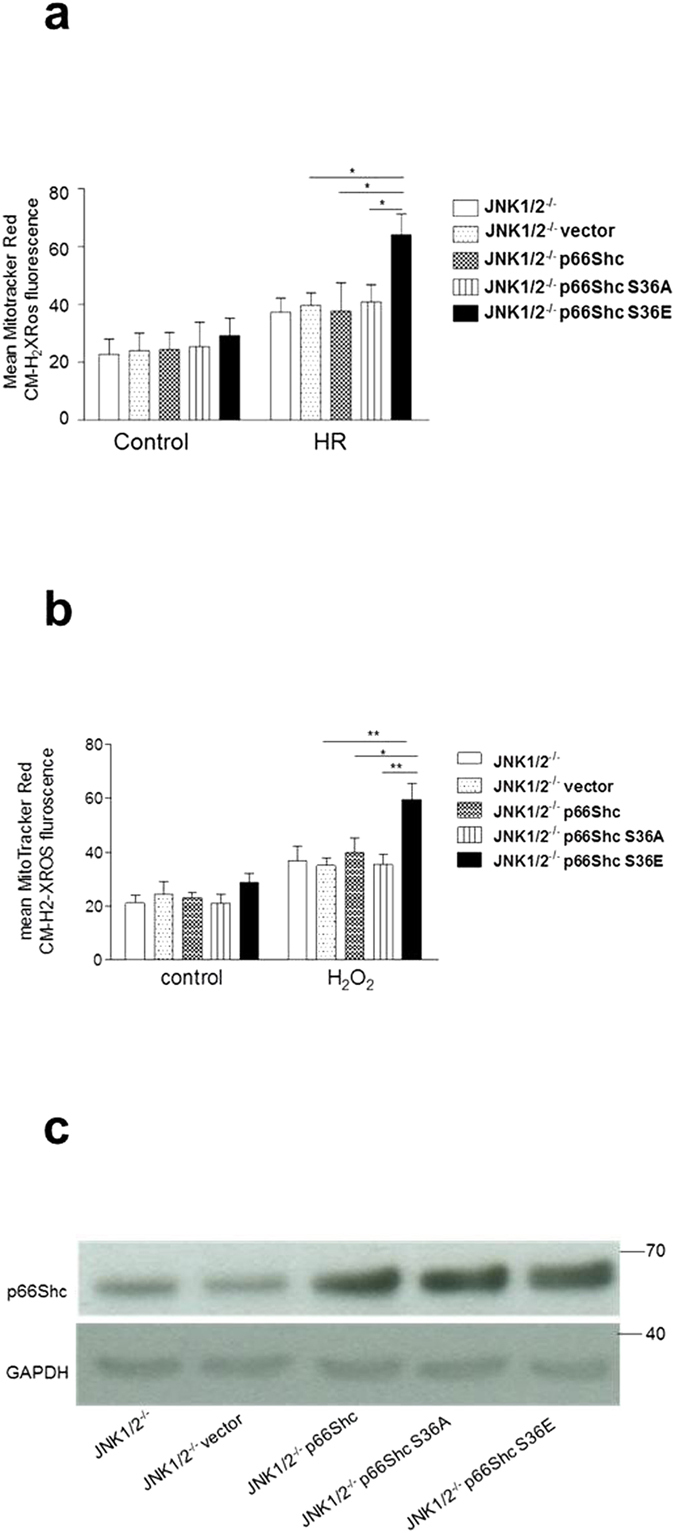
Low mitochondrial ROS phenotype of JNK1/2^−/−^ MEFs is reversed by overexpression of p66ShcS36E mutant protein. Mitochondrial ROS were detected by MitoTracker Red CM-H_2_XRos dye following treatment with HR (hypoxia 90 min, reoxygenation and staining 30 min) (**a**) or H_2_O_2_ (1 mM, 30 min) (**b**). Overexpression of p66Shc was confirmed by western blotting (**c**). Each experiment was repeated at least three times. Statistical analysis was performed using ANOVA (**p < 0.01, ***p < 0.001).

To substantiate the role of S36 phosphorylation by JNK1/2 in ROS production we tested whether expression of a p66Shc mutant with mutation of S36 to glutamic acid (S36E) will rescue ROS production following prooxidant treatment or HR. MEF JNK1/2^−/−^ were stably transfected with the appropriate expression constructs and analyzed for protein expression (Fig. 4c). Expression of the acidic exchange mutant but not the S36A mutant expressed at equal levels, resulted in significantly increased ROS production (Fig. 4a,b), confirming the importance of this site for ROS production under the conditions studied here and supporting a role for JNK1/2 in p66Shc S36 phosphorylation – ROS axis. The p66Shc S36 mediated regulation of ROS in JNK1/2 ^−/−^ MEFs was further confirmed by staining cells after stress with DCF-DA and analysis with FACS (Supplementary Figure S3)

### PKCß does not function upstream of JNK1/2

**Figure 5:**
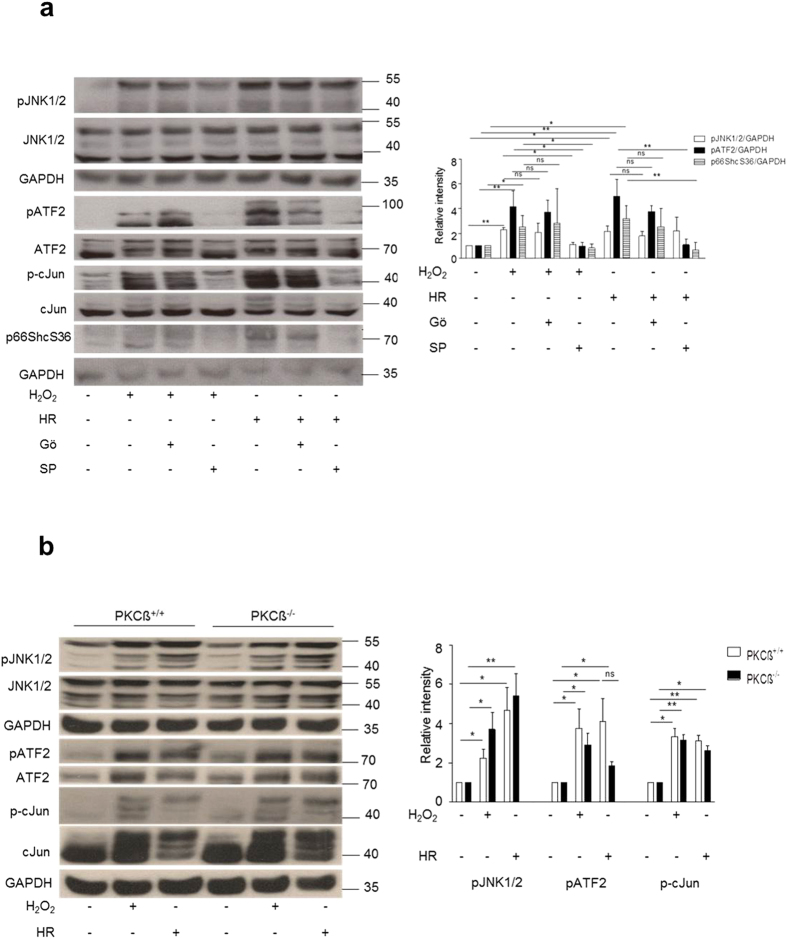
PKC is not the upstream kinase of JNK. JNK^+/+^ MEFs were treated either with Gö6976 (1 μM) or SP600125 (20 μM) both under HR and H_2_O_2_ treatment. Phosphorylation of JNK or its substrates c-Jun and ATF2 was detected by Western blotting (**a**). Pattern of phosphorylation of JNK under H_2_O_2_ and HR was confirmed in PKCß^+/+^ or PKCß^−/−^ MEFs (**b**). Representative blots and summary graphs as mean ± SD for at least three individual experiments are shown (*p < 0.05, **p < 0.01, ***p < 0.001).

As it is already been shown that PKCß regulates p66ShcS36 phosphorylation under different stress conditions, we next sought to determine if PKC acts as upstream kinase of JNK and for this we used Gö6976 and SP600125 upon prooxidant treatment and HR in MEFs. Our data show that there is no effect on JNK phosphorylation upon H_2_O_2_ treatment in the presence of Gö6976 and only a marginal effect on JNK phosphorylation upon HR. Under both conditions p66ShcS36 phosphorylation was completely abolished by the JNK inhibitor (Fig. 5a). The same results were observed when PKCß wild type and knockout cells were compared (Fig. 5b).

### p66Shc and JNK interact physically and recombinant JNK1 but not PKCß phosphorylates p66ShcS36

**Figure 6:**
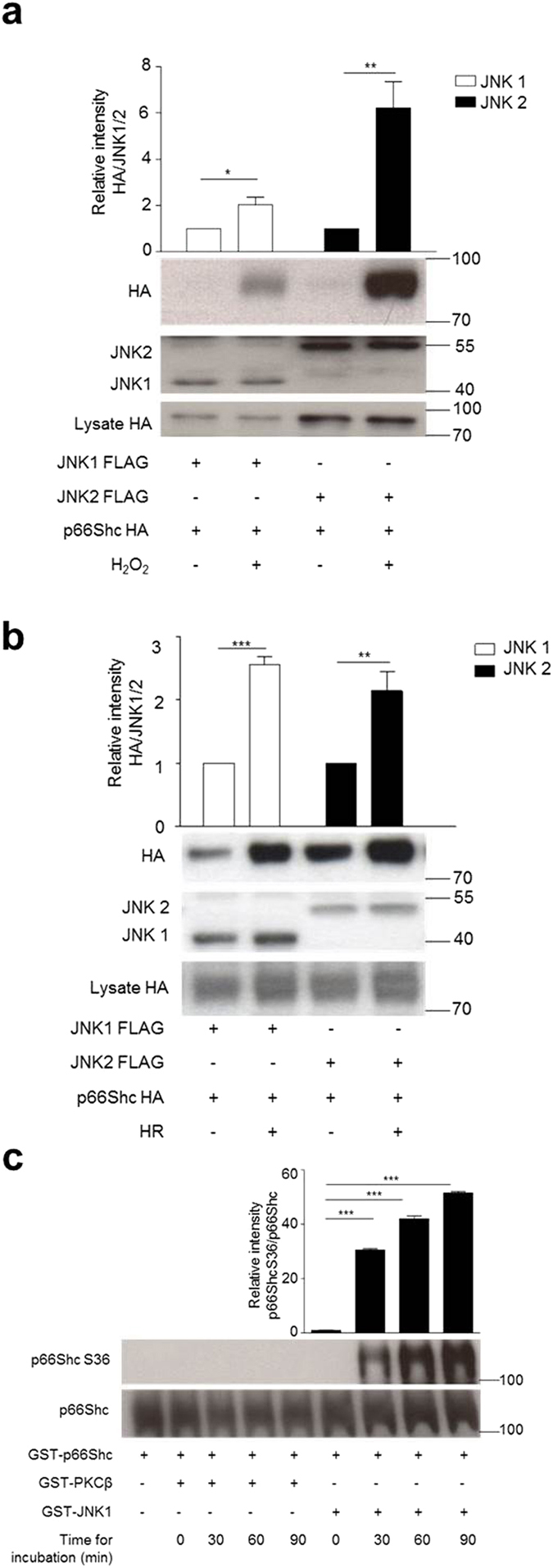
JNK1/2 and p66Shc interaction. An expression construct for HA-tagged p66Shc together with either JNK1 or JNK2, both carrying a FLAG tag, were transiently transfected into HEK293 cells (**a,b**). Cell lysates were subjected to immunoprecipitation with antibodies directed against the FLAG tag and associated p66Shc was visualized with an antibody directed against the HA-tag. Both, untreated and cells stressed with H_2_O_2_ (1 mM, 15 min) (**a**) or hypoxia 8 hours (**b**) were analyzed (n ≥ 4). Equal expression of JNK and p66Shc was confirmed by HA and JNK antibody (Below) while fold changes in densitometric band intensities, acquired by image J were compared. Band intensity of control sample was taken as 1 (Above) and statistical analysis was performed using *t*-test. Recombinant PKCß and JNK were used to phosphorylate bacterially expressed p66Shc under defined conditions. Phosphorylation was monitored by p66ShcS36 antibody (**c**) while fold changes in densitometric band intensities, acquired by image J were compared. Band intensity of control sample having only p66Shc protein without kinases was taken as 1 (n = 3) and statistical analysis was performed using ANOVA.

To obtain further support for a crosstalk between JNK and p66Shc in the regulation of p66Shc activation we checked for direct interaction by performing co-immunoprecipitation assays. Both, an HA-tagged version of p66Shc and FLAG-tagged constructs for either JNK1 or JNK2 were expressed in HEK293 cells and a possible association was tested by immunoprecipitating FLAG-tagged JNK with anti-FLAG M2 affinity gel. As shown in Fig. 6, treatment with 1 mM H_2_O_2_ (a) or 8 h of hypoxia (b) resulted in increased association of both proteins. These data demonstrate that association occurs, which depends on the presence of a stress stimulus. Finally recombinant PKCß and JNK were used to phosphorylate bacterially expressed p66Shc under defined conditions. Phosphorylation was monitored by p66ShcS36 antibody. As shown in Fig. 6c, in contrast to PKCß, JNK efficiently phosphorylated S36 and cJun, which was used as positive control (data not shown). In search for other possible JNK phosphorylation sites p66Shc tryptic digests of p66Shc were analyzed by mass spectrometry. We failed to detect any other potential site with increased phosphorylation over time (data not shown).

### Linking cell death and damage to JNK1/2-regulated ROS production

**Figure 7:**
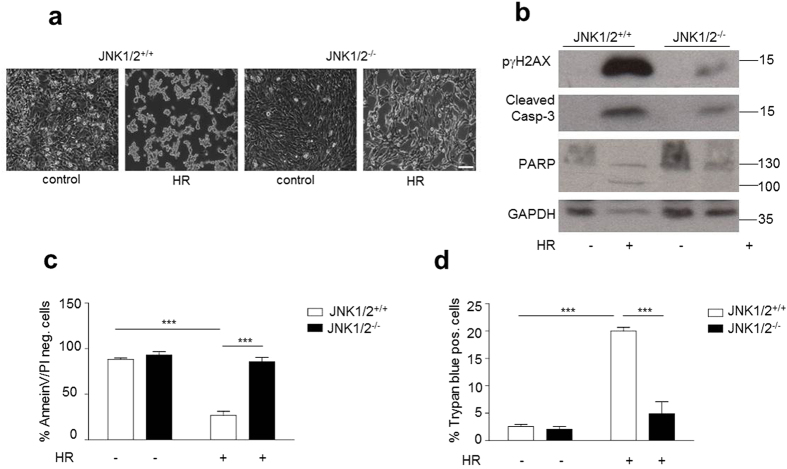
JNK1/2 deficiency results increased resistance to cell death induced by HR. JNK1/2^+/+^ and JNK1/2^−/−^ MEFs were subjected to HR (hypoxia 3 h, reoxygenation 16 h). Morphological changes were documented under the phase-contrast microscope (**a**, 100× magnification) and cell death was assessed by trypan blue (**d**) or Annexin V/PI staining followed by FACS analysis (**c**). Immunoblotting was performed to analyze the phosphorylation of histone γH2AX and PARP/Casp-3 cleavage (**b**) (n ≥ 4). Size bar = 100 μM. Statistical analysis was performed using ANOVA (***p < 0.001).

**Figure 8:**
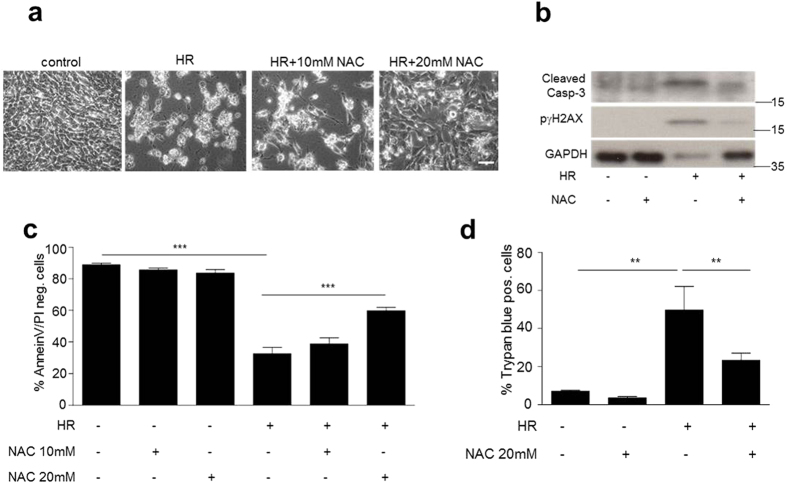
HR induced cell death in JNK^+/+^ MEFs is ROS mediated. Cells were pretreated with 5 or 10 mM of the antioxidant N-acetyl-cysteine (NAC) for 1 h prior to HR (hypoxia 3 h, reoxygenation 16 h). Phase-contrast images are shown (**a**, 100× magnification). Cell death or damage (**b**–**d**) was assessed as described in the legend to Fig. 6. Each experiment was performed at least three times. Size bar = 100 μM. Statistical analysis was done using ANOVA (**p < 0.01, ***p < 0.001).

Following the demonstration of a link between JNK activation and p66ShcS36 phosphorylation we went to address its role in cell damage and death. For this we used the hypoxia/reoxygenation protocol (HR) described in Material and Methods. While in JNK1/2^+/+^ MEFs this treatment resulted in the detachment and rounding up of cells, no such effect was observed in JNK1/2-deficient cells (Fig. 7a). This protective effect of JNK1/2 ablation was further confirmed when cell death was quantified by determining trypan blue uptake (Fig. 7d), Annexin V/PI staining pattern by FACS (Fig. 7c), caspase 3 and PARP cleavage (Fig. 7b). In both instances JNK1/2 deficiency protected cells from the effect of HR. As shown in Fig. 7b HR resulted in pronounced phosphorylation of γH2AX, which was much less pronounced in JNK1/2-deficient cells. A direct causative role of ROS in the killing of cells under the HR was investigated through the use of the antioxidant N-acetyl-cysteine (NAC), which prevented dose-dependently the HR-induced morphological alterations (Fig. 8a) as well as cell death (Fig. 8c,d) and γH2AX phosphorylation (Fig. 8b).

## Discussion

While the contribution of excessive ROS production to the pathogenesis of many diseases is realized, approaches for the clinical management of redox stress are unsatisfactory. In particular the use of antioxidants has been faced with only moderate clinical benefits^4^,^5^. This may be owed to the technical difficulty to timely and efficiently target antioxidants to the site of action of these short-lived compounds, which cause damage at narrow distance. Primarily mitochondrial ROS generated at the electron transport chain (ETC) located on the inner mitochondrial membrane during the process of oxidative phosphorylation (OXPHOS) have been implicated in the generation of oxidative damage during IR. Electron leakage at complex I and complex III leads to partial reduction of oxygen to form superoxide^44^. p66Shc has been shown to directly oxidize cytochrome *c* leading to the production of H_2_O_2_^26^. While major efforts are undertaken to develop protective antioxidants with efficient detoxification in the mitochondria (e.g.^45^,^46^), we and others are pursuing a strategy, which aims at limiting or preventing excessive ROS levels, preferentially by targeting the production of ROS.

In the work presented here we show that in a cellular model of ischemia/reperfusion the stress kinases (JNK, p38) get activated along with enhanced p66ShcS36 phosphorylation. We go on to demonstrate that S36 phosphorylation by JNK1/2 is critical for unleashing the pro-oxidant and pro-apoptotic function of p66Shc. p66Shc possesses oxidoreductase activity and causes oxidative damage in many disease settings^29^,^47^. The ablation of p66Shc had a similar cardioprotective effect^48^ than experimentally inhibiting complex I of the ETC^49^. Also eliminating ROS derived from non-mitochondrial sources like the NADPH oxidases (NOX) resulted in decreased tissue damage during IR in various settings. However, p66Shc may be a preferred target for intervention, because lack of p66Shc was compatible with normal development and adult survival^26^, while in the case of NOXs efficient inhibition may be faced with negative side effects. This has been demonstrated for NOX4, where a protective effect was observed against myocardial IRI following the knockout but additionally knocking down NOX2 actually increased the infarct area, most likely due to an requirement of low ROS signaling for protection^50^.

Published work stressed the importance of S36 phosphorylation in the activation of p66Shc. Work by the group of Rizzuto showed for the first time that PKCß may be the kinase phosphorylating this site^30^. Phosphorylation was thought to be required for PIN1 binding and mitochondrial import of p66Shc^26^,^29^. However, the sequence surrounding S36 predicts phosphorylation by proline-directed kinases and not PKC^32^,^33^,^34^,^35^,^36^. MAPKs have been implicated in the phosphorylation of S36^37^,^38^,^39^ and activation of JNK, p38 and ERK is commonly observed during ischemia and particularly early reperfusion^21^,^22^,^37^,^38^,^39^,^51^. A possible link between S36 phosphorylation by JNK and ROS production in response to various stimuli including UV radiation has been described^37^,^38^,^39^. Using more specific PKC inhibitors and cells deficient in PKCß under the experimental conditions studied here we failed to confirm the role of PKCß in phosphorylating S36 of p66Shc, while inhibition of PKCß or its deletion clearly affected ROS production to an extent observed with p66Shc deficient cells (Haller, Khalid *et al.* manuscript in preparation). The evidence that JNK1/2 phosphorylation of this site is critical is based on inhibitor experiments, but also on the use of knockout cells, and most convincingly shown by the use of rescue mutant, where the phosphorylation site was exchanged to a glutamic acid (S36E).

We also addressed the question whether PKC may function upstream of JNK1/2 as proposed previously^39^. The experiments performed here suggest that JNK activation is not controlled by PKC (Fig. 5a,b) and thus the question remains if PKC and JNK may be jointly required to regulate p66Shc activation, e.g. by targeting different phosphorylation sites, or if they are part of two separate scenarios for p66Shc activation. Both kinases get activated under the conditions studied here and we have recently identified and functionally characterized several PKC phosphorylation sites located in the PTB domain of p66Shc (Haller, Khalid *et al.* manuscript in preparation), which may be required for p66Shc activation under the conditions described here. The situation may be even more complex, as we demonstrate interaction of JNK1/2 with p66Shc (Fig. 6a,c), Pin1 has been implicated in the activation of JNK^52^, and Sab has been identified as mitochondrial binding partner for JNK under stress^53^. Future studies will have to clarify the individual contribution of these phosphorylation events and protein-protein interactions to the activation of p66Shc. Also other PKC family members have been proposed to function in the regulation of mitochondrial events with no connection to p66Shc yet. Activation of PKCε before ischemia protects mitochondrial function, while activation of PKCδ during reperfusion induces cell death^20^. In both cases the effects are also on ROS levels^54^,^55^,^56^.

In summary our data suggest that JNKs provide suitable targets for therapeutic intervention in conditions of oxidative damage. Although no clinical studies with JNK inhibitors have been reported so far^57^ these may become feasible soon as novel compounds for the inhibition of JNK1/2 keep being developed^58^.

## Material and Methods

### Cell culture

Wild type (wt) mouse embryonic fibroblasts (MEFs) or MEFs deficient in p66Shc^26^, JNK 1 and 2^59^ or PKCß^60^ were maintained in DMEM containing 10% fetal calf serum (FCS), 200 mM L-glutamine, penicillin (100 U/ml)/streptomycin (100 μg/ml) (all from PAA Laboratories, Pasching, Austria) at 37 °C, 5% CO_2_ in humidified atmosphere.

### Cloning and site-directed mutagenesis

For the generation of mutant p66Shc constructs, the following primers were used: p66Shc S36A (Forward: CCGGAGGAGCTGCCTGCCCCATCAGCTTCATCC, Reverse: GGATGAAGCTGATGGGGCAGGCAGCTCCTCCG), p66Shc S36E (Forward: CCGGAGGAGCTGCCTGAGCCATCAGCTTCATCC and Reverse: GGATGAAGCTGATGGCTCAGGCAGCTCCTCCG) (Eurofins MWG Operon Ebersberg, Germany). Site-directed mutagenesis was performed using the QuikChange II Site-directed mutagenesis kit from Stratagene (La Jolla, CA, USA). Amino acid exchanges were confirmed by DNA sequencing (Microsynth AG, Balgach, Switzerland).

### Plasmid DNAs, transfection procedure

The following plasmids were used in this study: pBABEpuro p66Shc (kindly provided by Marco Giorgio), pcDNA3 FLAG JNK1a1 (ID 13798), pcDNA3 FLAG JNK2a2-(ID 13755) (Addgene, Cambridge, MA), and pRec_p66Shc-HA-His (GeneCopoeia, Maryland, USA), pHyPer-dMito/pLenti6/V5-DEST Gateway lentiviral vector (kindly provided by Pidder Jansen- Dürr)^61^. MEF JNK1/2^+/+^, JNK1/2^−/−^ (3.5 × 10^5 ^cells/well) and HEK293 cells (6.5 × 10^5^) were transfected using Lipofectamine (Invitrogen, Eugene, OR, USA). Transiently transfected cells were used 48 hours (h) after transfection while stable cells lines were produced by appropriate antibiotic selection.

### Co- immunoprecipitations

HEK293 cells were transiently transfected with equal amounts of pcDNA3 FLAG JNK1a1 or pcDNA3 FLAG JNK2a2 and pRec_p66Shc-HA-His. 48–72 h after transfection, cells were lysed in 1 ml NP-40 lysis buffer (25 mM Trizma base, 150 mM NaCl, 10 mM Na_4_P_2_O_7_, 25 mM glycerol-2-phosphate, 10% glycerol, 0.75% NP-40, 25 mM NaF) supplemented with Protease Inhibitor Cocktail Set (Merck, Darmstadt, Germany). 40 μl of the lysate were mixed with 8 μl 6 x Laemmli buffer and incubated for 5 minutes at 95 °C to be used for expression control. Pre-clearing was performed with 40 μl Protein G Agarose beads (Roche, Mannheim, Germany) or mouse IgG agarose (Sigma Aldrich, Dorset, UK Sigma) and incubated for 1 hour at 4 °C, shaking at 300 rpm. Immunoprecipitation was done for 3 h with 40 μl anti FLAG M2 affinity gel (Roche Diagnostic, Vienna, Austria). Proteins were isolated by boiling with 2 x Laemmli buffer for 5 minutes and were detected with immunoblotting.

### Hypoxia Reoxygenation (HR), simulated ischemia reperfusion (sIR) and prooxidant treatment

MEFs (3 × 10^5^) or HL-1 (6.5 × 10^5^) cells/well were subjected to hypoxia/reoxygenation and HL-1 with sIR as previously described^22^. Briefly, MEFs or HL-1 cells were washed with PBS and incubated with 1 ml of 0.5% medium or simulated ischemia buffer (125 mM NaCl, 8 mM KCl, 1.2 mM KH_2_PO_4_, 1.25 mM MgSO_4_, 1.2 mM CaCl_2_, 6.25 mM NaHCO_3_, 20 mM 2-deoxyglucose, 5 mM sodium lactate, 20 mM HEPES, pH 6.6), respectively, in a hypoxia chamber (Billups-Rothenberg). Oxygen concentration was reduced to 0.5% by flushing the oxygen with nitrogen (N_2_) and was maintained for 1–6 h controlled by an oxygen sensor (Dräger, Lubeck, Germany). For reoxygenation starvation medium was replaced with full serum medium or reperfusion buffer (110 mM NaCl, 4.7 mM KCl, 1.2 mM KH_2_PO_4_, 1.25 mM MgSO_4_, 1.2 mM CaCl_2_, 25 mM NaHCO_3_, 15 mM glucose, 5 mM sodium lactate, 20 mM HEPES, pH 7.4) and cells were incubated in normoxic culture conditions. Inhibitors for PKC (Gö6976, Calbiochem, Vienna, Austria), p38 (BIRB796, Axon Medchem, The Netherlands), MEK (UO126, Promega, Madison, USA) and JNK (SP600125, LC Laboratories, MA, USA) were applied 1 h prior to stress application and maintained during stress conditions. H_2_O_2_ were obtained from Sigma-Aldrich (St. Louis, MO, USA) and directly added to the culture medium at the final concentrations indicated.

### ROS measurements

Mitochondrial reactive oxygen species (ROS) were imaged by fluorescence microscopy after staining the cells with 100 nM MitoTracker Red CM-H_2_XRos (Life Technologies, Paisley, UK) in serum-free DMEM as described previously^22^. Briefly, (3 × 10^4^–6 × 10^4^) cells per well were seeded in 8-well chambered Lab-Tek cover glasses (Nalge Nunc, Rochester, NY, USA). Cells were stained for 30 minutes at 37 °C after stress application according to experimental settings. Images were taken using an Olympus IX-70 inverted microscope. Grey values were measured using Scion Image software (Sigma-Aldrich, St. Louis, MO, USA) for Windows. For the detection of mitochondrial H_2_O_2_ levels we also used a protein-based detection system. Cells were stably or transiently transfected with pHyPer-dMito/pLenti6/V5-DEST Gateway lentiviral vector. Fluorescence was detected by confocal microscopy^23^ and quantitative analysis was done using FACSCalibur (BD, Franklin Lakes, NJ, USA).

### Immunoblotting

Proteins were isolated and detected as described previously^23^,^24^,^62^. Primary antibodies raised against following proteins were used: phospho-p38 MAPK (9212), phospho-MAPKAP kinase 2 (MK2) (3044), MAPKAP kinase 2 (3042), phospho γH2AX (9718), phospho-ATF2 (9221) from Cell Signaling Technology, Boston, MA), p38MAPKα (sc-535), phospho ERK (sc-16982R), ERK1 (sc-94), JNK (sc-571), ATF2 (sc-187) from Santa Cruz Biotechnology, Santa Cruz, CA), pJNK (AF1205), R&D Systems Minneapolis, MN, USA, GAPDH (AM4300, Ambion, Grand Island, NY), a-tubulin (T5168, Sigma Aldrich, Dorset, UK), Shc1 (610879), BD Biosciences, San Diego, CA, USA, pSer36-Shc1 (54518, Abcam, Cambridge, UK) and anti-HA-Peroxidase (12013819001), Roche, Mannheim, Germany. Antibodies were visualized by ECL Western blot detection reagents (Amersham, Buckinghamshire, UK), quantified by densitometric scanning using the image J program (NIH, Bethesda, MD) and normalized against loading control.

### Cell Death Detection

7 × 10^5^ JNK1/2^+/+^ or JNK1/2^−/−^ MEFs were seeded in 6 well plates (BD, Vienna, Austria) for 24 h before stressing them with hypoxia (3 h) and reperfusion (16 h, unless otherwise stated). Apoptosis was determined using FACS after staining with Annexin V-FITC (Enzo Lifesciences, Farmingdale, NY, USA) and propidium iodide (Carl Roth, Karlsruhe, Germany). Cells were collected, centrifuged and incubated for 10 minutes on ice in 30 μl Annexin staining solution, containing 1.5 μg propidium iodide and 1 μg Annexin V-FITC. 400 μl Annexin binding buffer (containing 10 mM HEPES, 140 mM NaCl and 2.5 mM CaCl_2_) was added and centrifuged. For FACS measurement cells were resuspended in 300 μl full-serum DMEM. The percentage of vital cells, Annexin V- and PI-negative, was determined using CellQuest software for FACSCalibur (BD, Franklin Lakes, NJ, USA). Cell viability was also determined by staining dead cells with trypan blue and counting unstained cells with a microscope. Briefly, after stress, cells were collected and centrifuged for 5 minutes at 200× g and resuspended in 200 ul 10% DMEM. 1:1 dilution was made with filter sterile trypan blue (Sigma Aldrich, Dorset, UK). Stained and unstained cells were counted by using Neubauer counting chamber. DNA damage and cell death was also monitored by using antibodies detecting phosphorylation of γH2AX, cleaved caspase 3 and PARP fragments respectively by western blotting.

### Kinase assay

Purified recombinant p66Shc-GST fusion protein and 100 ng recombinant GST-JNK1 (Sigma Aldrich, Dorset, UK) or PKCß were incubated in 50 μl of kinase assay buffer (200 mM Tris-HCl pH 7.5, 100 mM MgCl2, 0.5 mg/ml BSA, 50 μM DTT, 50 μM riboATP; and PKCßII Kinase Enzyme System, Promega, Madison, USA, respectively) for 15, 30 and 90 minutes at 30 °C. The reaction was stopped by adding 5 × SDS-PAGE sample buffer and proteins were resolved on SDS-PAGE gels and probed by Western blotting with p66ShcS36 and total p66Shc antibody.

### Statistics

All data are presented as mean ± SD using *t*-test or ANOVA. Statistical analysis was done using GraphPad Prism 5 (GraphPad Software, La Jolla, CA, USA). Significance values were designated as follows: *p < 0.05, **p < 0.005, ***p < 0.0005.

## Additional Information

**How to cite this article**: Khalid, S. *et al.* cJun N-terminal kinase (JNK) phosphorylation of serine 36 is critical for p66Shc activation. *Sci. Rep.* **6**, 20930; doi: 10.1038/srep20930 (2016).

## Supplementary Material

Supplementary Information
