# Supplementary material for: cJun N-terminal kinase (JNK) phosphorylation of serine 36 is critical for p66Shc activation
Source: Sci Rep. 2016 Feb 12;6:20930. doi: 10.1038/srep20930 (PMC4751440; doi:10.1038/srep20930)
Supplement: Supplementary Information [file srep20930-s1.pdf]

Supplementary Information

cJun N-terminal kinase (JNK) phosphorylation of serine 36 is critical for p66Shc activation

Sana Khalid, Astrid Drasche, Marco Thurner, Martin Hermann, Muhammad Imtiaz Ashraf, Friedrich Fresser, Gottfried Baier, Leopold Kremser, Herbert Lindner, Jakob Troppmair

Running title: JNK1/2 control p66Shc activation

Supplementary Figure S1: Inhibition of JNK kinases prevents p66ShcS36 phosphorylation upon prooxidant treatment and HR.

pP38, P38 and GAPDH are shown for Figure 1a (a) and for Figure 1b (b). The lanes marked by an open box are shown in Figure 1b.

Supplementary Figure S2: JNK inhibition prevents p66ShcS36 phosphorylation upon sIR in HL-1 cardiomyocytes.

HL-1 cardiomyocytes were subjected to 45 minutes of ischemia and 15 minutes of reperfusion. Cell were either lysed to probe with antibodies for p66ShcS36 (a), stained with MitoTracker Red CM-H_2XRos fluorescent dye (n≥4) (b) or immunoblotted with antibody against phospho-γH2AX (c). Representative blots and a summary graph of at least three individual experiments are shown (panels a, c). Statistical analysis was done using ANOVA (*p<0.05 **p<0.01).

Supplementary Figure S3: JNK1/2$^{-/-}$ MEFs overexpressing p66ShcS36E mutant protein show increased ROS levels upon stress

JNK1/2$^{-/-}$ MEFs overexpressing p66Shc mutants were stressed with H_2O_2 (1mM, 15min) (a) or hypoxia 90 minutes (b). After trypsinization cells were stained with DCF-DA 7µM for 15 minutes and analyzed with FACS. Statistical analysis was done using ANOVA (*p<0.05 **p<0.01, ***p<0.001).

Supplementary Figure S4: JNK1/2 deficiency results in an impaired p66ShcS36 phosphorylation upon HR and prooxidant treatment

Absence of JNK 1/2 in MEFs is confirmed with western blotting (a). The lanes marked by an open box are shown in main Figure 2a (b) and 2b (c).

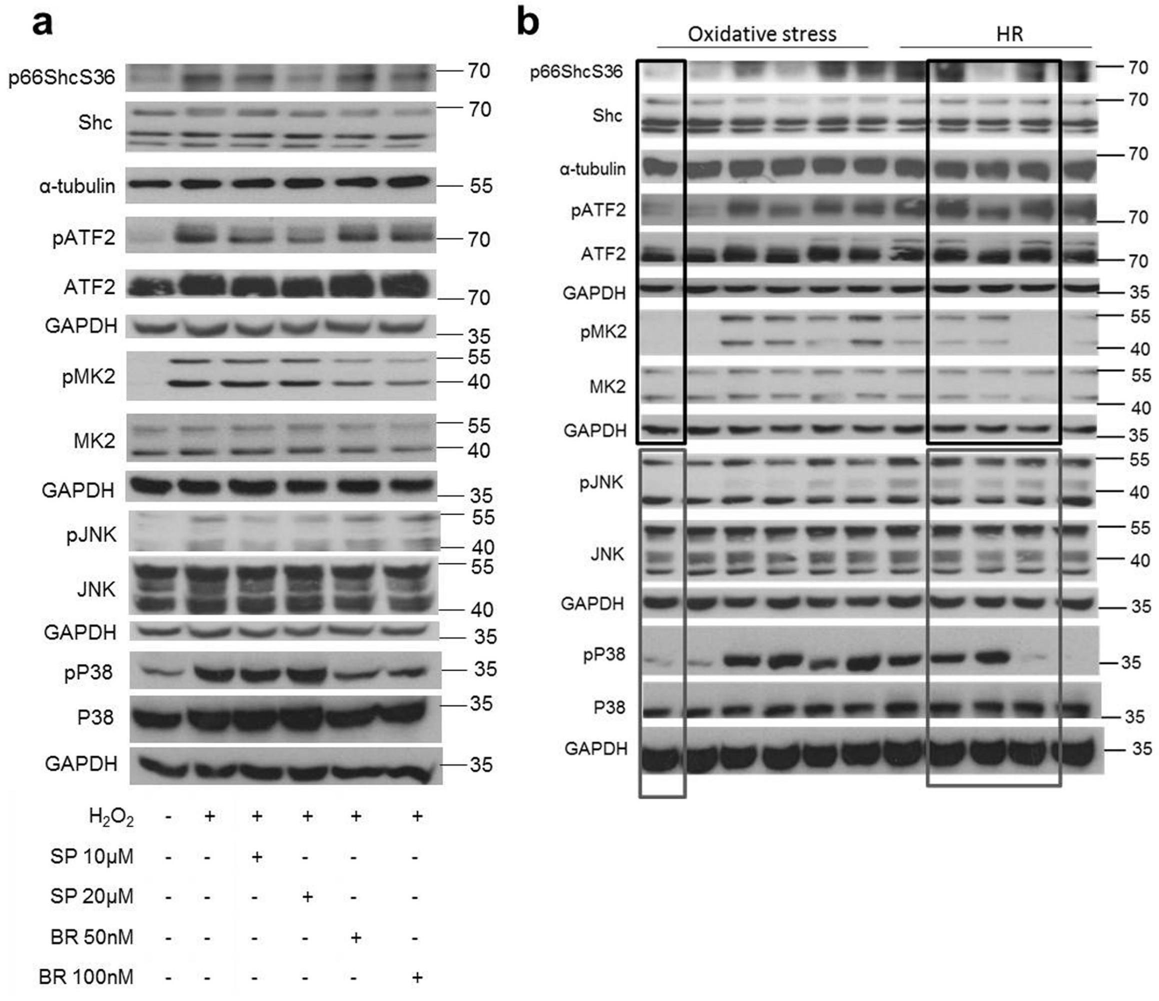

Supplementary Figure S1

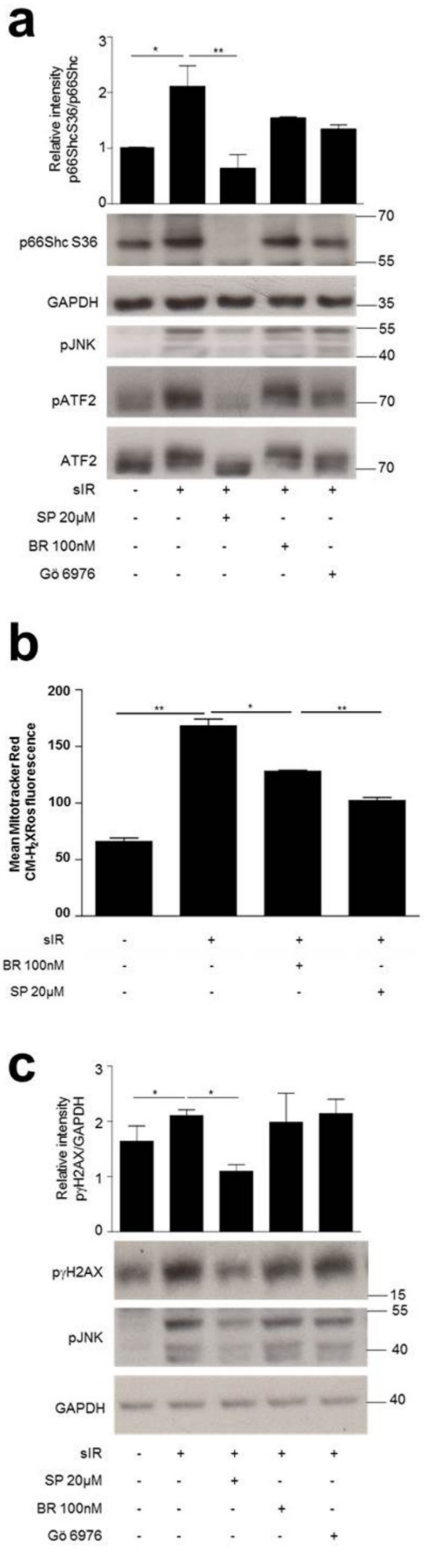

Supplementary Figure S2

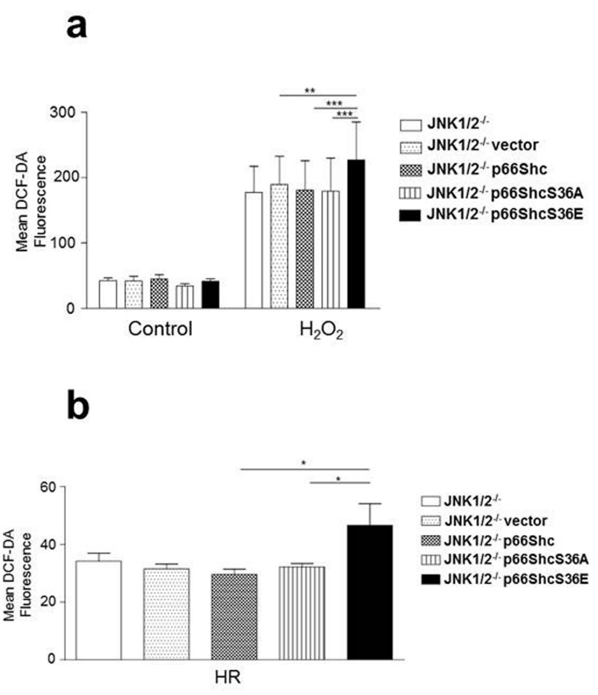

Supplementary Figure S3

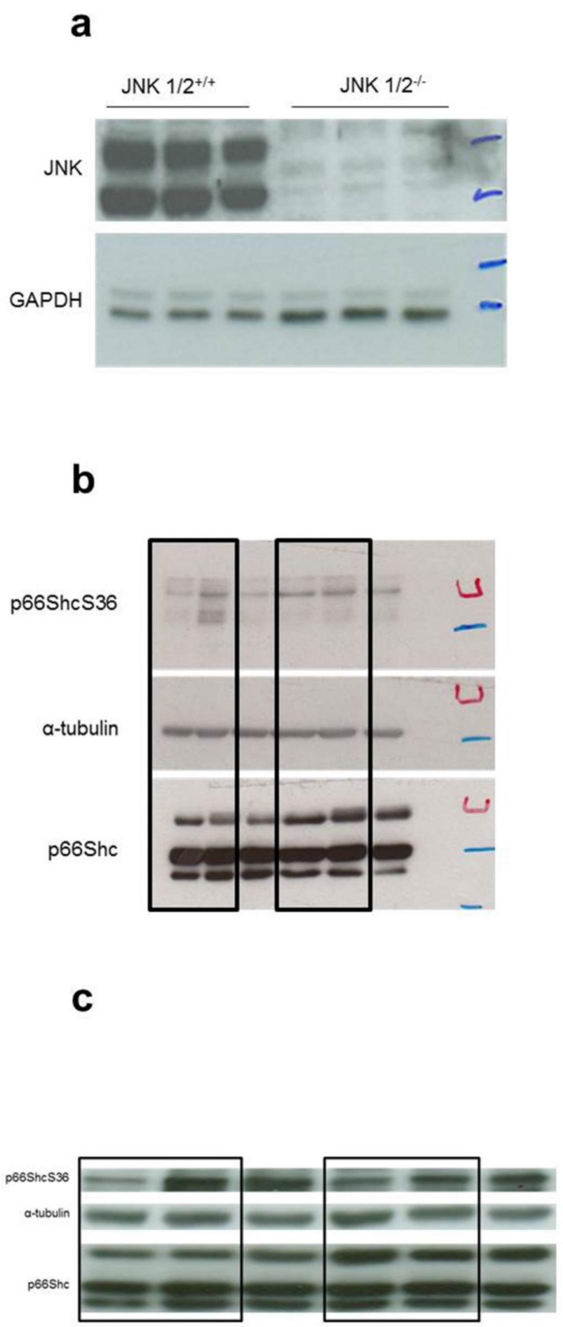

Supplementary Figure S4
